# Using the Generic Model of Psychotherapy to Develop a Culturally-Sensitive Approach to Psychotherapy With Sexual and Gender Minority Patients

**DOI:** 10.3389/fpsyg.2020.599319

**Published:** 2020-12-09

**Authors:** Alemka Tomicic, Claudio Martínez, Juliana Rodríguez

**Affiliations:** Center for Studies in Clinical Psychology and Psychotherapy, Faculty of Psychology, Universidad Diego Portales, Santiago, Chile

**Keywords:** generic model of psychotherapy (GMP), LGBT patients, psychotherapy research, affirmative interventions, cultural sensitive psychotherapy

## Abstract

This article discusses how the Generic Model of Psychotherapy (Orlinsky and Howard, 1987) can help to organize the psychotherapy research and the knowledge in the field of psychotherapy for sexual and gender minority patients. The structure that this traditional model provides is a good foundation for research in this field, inasmuch as it stresses macrosocial aspects that determine the provision of psychotherapy and contextualize its outcomes. Each one of the main components offered by the Generic Model of Psychotherapy – Determinants, Processes, and Consequences – are specified for psychotherapy with LGBT patients and are illustrated with a selection of already existing research in the field of sexual minorities; they are also stress areas marked by knowledge gaps that require future developments. In addition, a set of questions are proposed to contribute to new studies, including the clinical implications that can be derived from this model.

## Introduction

Globally, studies conducted over the last 25–30 years have yielded a body of knowledge on psychological interventions sensitive to the specificities of working with LGBT patients (King et al., 2007; American Psychological Association, 2015; O’Shaughnessy and Speir, 2018). This article argues for the use of the Generic Model of Psychotherapy (Orlinsky and Howard, 1987) as an organizing framework for the psychotherapy research and the knowledge produced in this field with sexual and gender minority patients. We suggest this framework as a map to identify areas in the field of psychotherapy with LGBT+ (Lesbian, Gay, Bisexual, Transgender, Intersex and other sexuality, sex and gender diverse) patients, where the generation of more learnings, as well as specific proposals for clinical development, are needed. Psychological interventions, the so-called affirmative psychotherapy model, and other proposals for clinical work and mental health support for LGBT+ people have gradually gained traction alongside a greater demand for treatment (e.g., O’Shaughnessy and Speir, 2018; Pereira et al., 2019). However, studies in this field have yet to produce a theoretical model for integrating existing research, developing new information, and guiding clinical practice. The Generic Model of Psychotherapy (Orlinsky and Howard, 1987), used for years in psychotherapy research to organize the evidence produced, could guide researchers as well as practitioners’ to understand and integrate the core elements that determine the therapeutic process; the model also highlights the aspects of the social and cultural context that constitute and are conditions of possibility for psychotherapy. In addition, the model takes into account that the consequences of psychological treatments beyond their direct impact on individuals, highlighting their influence on patients’ family, social, or economic environment. These aspects, which contextualize psychotherapeutic treatments, are essential when considering psychotherapy research and practice with sexual and gender minority patients.

## The Generic Model of Psychotherapy

The Generic Model of Psychotherapy was originally devised as a transtheoretical framework, integrating a variety of available empirical findings about the therapeutic process and its outcomes (Orlinsky and Howard, 1987). The Generic Model emerged not as a theory of clinical practice, but as a conceptual model for guiding process-outcome psychotherapy research (Orlinsky, 2009). Specifically, it provides a framework for organizing the production of knowledge about the relationship between aspects that determine and constitute the psychotherapeutic process, along with its consequences on patients and their social environment. Orlinsky and Howard (1987) established a diagram with three main components that operate as a general taxonomy of psychotherapeutic activity: Determinants, Process components, and Consequences.

Determinants are the social and human contexts that precede and influence the psychotherapy; it also includes where and how psychotherapy occurs, including social and cultural elements (e.g., beliefs and attitudes to psychotherapy and mental health) as well as institutional aspects (e.g., public or private health care and available health insurance). They also concern the aspects linked to the therapist’s life and personality (e.g., life story, education, professional status, and career) and elements related to the patient (e.g., sociodemographic characteristics, life history, and current living conditions; Orlinsky and Howard, 1987; Orlinsky, 2009). The Process components describe the six dimensions of the psychotherapeutic process that are shared by most psychotherapeutic approaches (Orlinsky et al., 1994). These dimensions include the therapeutic contract, psychotherapeutic operations, the therapeutic bond, the participants’ openness toward the relationship, the intra-session impacts of psychotherapy, and the temporal patterns of treatment. Lastly, Consequences comprise the clinical outcomes of psychotherapy for patients and how participating in a psychotherapy process influences the patient’s life situation. Also, it includes psychotherapy influence on the professional trajectory and the life of the therapist. Finally, the consequences consider the impact on the proximal and contextual setting, where the psychotherapy takes place (Orlinsky and Howard, 1987; Orlinsky, 2009).

A quick review of the classical and current literature on psychotherapy research shows that the Generic Model of Psychotherapy is generally cited and/or categorized as one that maps and identifies the variables of the psychotherapeutic process; this is what these authors label as Process components (see Flückiger et al., 2018). However, fewer studies have addressed the other generic aspects of the model: the determinants and the consequences. For instance, in regards to the determinants, authors usually focus on the personality traits of the participants (e.g., Lingiardi et al., 2018); as for the consequences of psychotherapeutic activity, publications tend to refer to how treatments diminish patients’ symptoms (e.g., McFarquhar et al., 2018).

In turn, the Generic Model has inspired empirical process-outcome research (e.g., Kolden, 1991; Kolden and Howard, 1992), lines of research such as the dose-response model and stages of change in therapy (e.g., Howard et al., 1986; Kopta et al., 1994), and studies on therapist influence on treatment (e.g., Lutz et al., 2007). The Generic Model has also inspired research in psychotherapy with specific populations. For example, one of the most recent and thorough applications of the model is the research on psychotherapy with people with traumatic brain injuries (Coetzer, 2007, 2009). In this field, authors have based their approaches on the notion that the damage suffered affects nearly all the aspects of patients’ existence, including their sense of self; consequently, they consider that a partial examination of psychotherapeutic process is insufficient. In this case, the model provides a general scheme that emphasizes elements that make it possible to combine approaches, theories, and techniques to be used with these patients. We believe that it can be similarly employed to organize and guide research on psychotherapy with LGBT patients.

## Applying the Generic Model for Researching Psychotherapy with LGBT+ Patients

### Determinants of Psychotherapy With LGBT+ Patients

As previously noted, in the Generic Model, determinants are contextual elements that directly or indirectly influence psychotherapeutic activity within a given field (Orlinsky, 2009). The core determinants are participants’ personality and identity before the therapy, beyond their roles as therapist/patient, including sexual orientation and gender. They also include the therapists’ professional status as well as their academic training and psychotherapy expertise. Lastly, determinants also include the institutional therapeutic approach adopted by the center, where people are treated and more collective aspects such as the community, the culture, and the social values that constitute the therapeutic setting.

**Figure 1:**
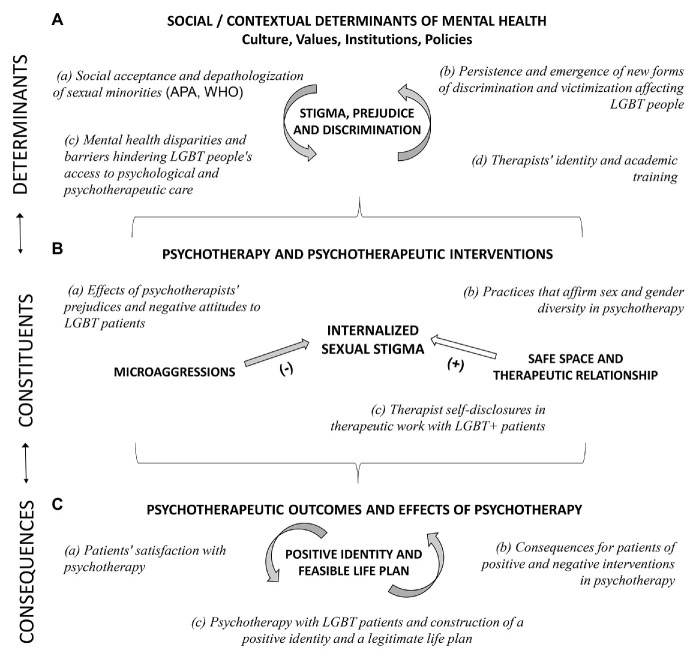
Generic model for researching psychotherapy with lgbt+ patients. **(A)** Determinants, **(B)** Constituents, and **(C)** Consequences.

As for the determinants of psychotherapy with sex- and gender-diverse people, the following aspects are particularly relevant in research and practice: (a) changes leading to the gradual acceptance and depathologization of gay, lesbian, bisexual, and transgender people, (b) the persistence and emergence of new forms of discrimination and victimization affecting LGBT people, (c) mental health disparities and barriers hindering LGBT patients’ access to psychological and psychotherapeutic care, and (d) therapists’ identity and training (see Figure 1A).

#### Social Acceptance and Depathologization of Sexual Minorities

From the late 1990s onward, society and culture have become increasingly open to recognize the rights of people who identify as lesbian, gay, bisexual, and trans (LGBT; Cáceres et al., 2008; Valfort, 2017). Social movements and political activists have promoted laws and major changes involving the social recognition, non-discrimination, and protection of the human rights of LGBT people (e.g., International Commission of Jurists, 2007; International Drafing Committe, 2017; Martínez et al., 2019a; Vial, 2019).

With respect to psychiatric and psychotherapeutic disciplines, there has been a positive but slow process of depathologization of sexual and gender diversity. The first edition of the Diagnostic and Statistical Manual of Mental Disorders (DSM), published by the American Psychiatric Association (APA) in 1952, classified homosexuality as a psychopathic personality disorder, which meant that people belonging to a sexual minority group were regarded not only as mentally diseased, but also as dangerous. Thanks to LGBT activism and depathologizing psychological research, the APA stopped considering homosexuality to be a mental disorder; however, the psychopathological category *egodystonic homosexuality* was introduced. Homosexuality was removed as a psychiatric diagnosis only in 1987, with the publication of the DSM-III-R (Drescher, 1998; Roughton, 2002). Transgenderism was included as a *gender identity disorder* in the DSM-III, published in 1973. In the fifth version of the manual (2013), this diagnostic category was renamed *gender dysphoria* in order to depathologize transgender people in terms of their identity and only keep it associated with a state of anxiety and discomfort (Bidell and Stepleman, 2017). Lastly, the World Health Organization (WHO) removed the gender non-conformity category as a mental disorder from the eleventh revision of the International Classification of Diseases (World Health Organization, 2018).

#### Persistence and Emergence of New Forms of Discrimination and Victimization Affecting LGBT People

Despite the great strides made in achieving social inclusion, depathologization, and the consequent destigmatization of sexual minorities, rejection, discrimination, and prejudice indexes remain high. In addition, more subtle or implicit forms of discrimination have emerged (Lingiardi et al., 2015; Kiebel et al., 2017; Vial, 2019) that continue to threaten the everyday lives of LGBT people and result in high stress levels for them (Barrientos et al., 2014). The studies published by Meyer (1995, 2003) on the Minority Stress Model have made it possible to generate a large body of evidence on how permanent victimization, discrimination, and homolesbotrans negativity cause LGBT people to display poorer mental health indicators than their heterosexual and cisgender peers (APA/Division 44, 2000; Meyer, 2003; Logie, 2012; Hatzenbuehler et al., 2013; Barrientos et al., 2014; Bidell and Stepleman, 2017). Specifically, the internalization of sexual stigma and the concealment of one’s diverse sexual identity constitute stressors that may be expressed through low self-esteem and self-loathing, causing distress around revealing one’s sexual orientation to others, self-exclusion and detachment from other LGBT individuals, a negative view of same-sex sexual activity, depression, and self-inflicted violence (Gillis and Cogan, 2009; Newcomb and Mustanski, 2010; Pereira and Rodrigues, 2015; Michaels et al., 2016; Tomicic et al., 2016, 2019; Austin and Goodman, 2017; Martínez et al., 2019b, Unpublished).

#### Mental Health Disparities and Barriers Hindering LGBT People’s Access to Psychological and Psychotherapeutic Care

In a systematic review of research on LGBT suicide and mental health spanning 10 years (2004–2014), it was concluded that the suicidality is a major problem in these populations, with suicidal behaviors reaching prevalence of 20–50%, up to seven times higher than those of heterosexual and cisgender people (Tomicic et al., 2016). In addition, it has been observed that LGBT people display high prevalence of mood disorders and anxiety (Cochran and Mays, 2000; Meyer et al., 2007; King et al., 2008) and problematic alcohol and drug use (Cochran and Mays, 2006; King et al., 2008; Institute of Medicine, 2011; Kelly et al., 2015). A mental health survey aimed at LGBTI+ people conducted in Chile revealed that 64.4% of the respondents had thought about committing suicide and 24.4% had made at least one attempt; also, it showed that 22% of the GB+ men and 34% of the GB+ women surveyed had symptomatology indicative of moderate to severe depression (Tomicic et al., 2019).

These mental health disparities suggest that LGBT+ people are more likely to request psychiatric and psychological care than their heterosexual and cisgender peers (Jones and Gabriel, 1999; Cochran and Mays, 2000, 2006). In line with this, evidence obtained in North America and some European countries indicates that LGB people use mental health services more often than heterosexual people. For instance, in Canada, between 2003 and 2005, high rates of health care service usage by LGB individuals were reported across all specializations, amounting to an increase of over 200% compared to heterosexual users (Tjepkema, 2008). In the United States, a study on mental health service usage (Cochran et al., 2003) found that more than half of gay and bisexual men had received some type of psychological or psychiatric treatment, and at a higher rate than heterosexual men. Likewise, two thirds of the lesbian and bisexual women surveyed and reported having used mental health services, also at a higher rate than their heterosexual peers. Recently, Platt et al. (2017) reported that, in the United States, the annual prevalence of mental health consultations with mental health professionals is 18.91% for gays and lesbians and 25.97% for bisexuals. More specifically, Murphy et al. (2002) found that more than half of the psychotherapists surveyed had worked with at least one sex- or gender-diverse patient during the last week. In Chile, a survey of 556 mental health professionals conducted between October 2017 and January 2018 showed that 42.5% were treating at least one sex- or gender-diverse patient, while 77% reported having done so at least once in their career (Martínez et al., 2018b). Also in Chile, a survey of mental health in LGBTI+ people conducted between September and October 2018 showed that 18.1% of the participants were in psychotherapy, while 64% stated that they had received psychological and/or psychotherapeutic treatment at least once. Of the 489 participants who reported having attended therapy at least once, 34.9% had consulted a professional due to mood disorders, 22.6% due to issues with their sexual orientation, and/or gender expression, 18.6% due to anxiety disorders, 12.4% due to relational problems, 8.1% due to suicidal behavior, 5.1% due to other disorders, 1.6% due to LGBT harassment, 4.9% due to mourning or separation, and 1% due to sexual violence (Tomicic et al., 2019). Another survey conducted in the same year, entitled Being a Lesbian in Chile (Ser lesbiana en Chile), indicated that 32.8% of the women surveyed had received psychological or psychiatric care specifically due to their sexual orientation (Agrupación Rompiendo el Silencio, 2019).

Barriers hindering LGBT people’s access to mental health and psychotherapeutic treatment are of three types: individual, related to the health care system, and associated with psychiatrists, psychologists, and psychotherapists (McIntyre et al., 2011). Regarding individual barriers, some studies suggest that internalized stigma, hypervigilance, anticipation and fear of discriminatory situations, and negative expectations of psychological and psychotherapeutic care constitute major access barriers affecting LGBT people (Avery et al., 2001; Tomicic et al., 2019). Thus, for instance, due to stigmatization, many patients decide not to reveal their sexual orientation or gender identity (D’Augelli and Grossman, 2001; Calton et al., 2016; Rossman et al., 2017) or refrain from seeking professional help (Calton et al., 2016; Bidell and Stepleman, 2017). Regarding barriers associated with the health care system, a study of mental health care service providers conducted in Canada revealed issues inherent to the medical care model, which tends to ignore the particular aspects of the social context of LGBT people seeking help, limited availability of mental health and psychotherapeutic services specifically aimed at LGBT people, and system-level disincentivization preventing practitioners from providing sex-diverse people with affirmative care (McIntyre et al., 2011).

#### Therapists’ Identity and Academic Training

Despite the epidemiological data available and the increase in psychological and psychotherapeutic consultations, studies conducted in the United States and Europe indicate that few mental health professionals have specialized in working with LGBT people (Grant et al., 2011; Rutherford et al., 2012; Bidell, 2016). Also, findings suggest that explicit or implicit prejudices and negative attitudes against LGBT people constitute a major barrier preventing sexual and gender minority groups from using mental health services (Bidell, 2016; Bidell and Stepleman, 2017). Similarly, for instance, a survey of mental health professionals in Chile (Martínez et al., 2018b) showed that 66.73% of the participants displayed a moderate to high level of prejudice against LGBT people and that psychotherapists’ training in sexual and gender diversity issues did not correlate with differences in terms of prejudice levels. However, it was found that practitioners who had a LGBT relative or close friend were significantly less prejudiced than those who did not have one.

The therapist’s cultural sensitivity – as a value incorporated into his/her/their personality – is another potential key determinant for psychotherapy. Research has shown that, when patients perceive that their therapists are attuned to their culture, they see them as more reliable and feel more comfortable during treatment. This has the potential to improve the therapeutic alliance and facilitate psychological well-being (Owen et al., 2011). Several studies have concluded that, in order to work effectively with LGBT patients, therapists must feel comfortable with their own sexual identities and be ready to examine their beliefs, feelings, and prejudices, comfort or discomfort with the patients, cultural differences, and knowledge about how these aspects can affect the therapist-patient relationship (American Psychological Association, 2012, 2015; Pereira et al., 2019).

In this context, training programs focused on the specific features of psychotherapy with LGBT people and increased cultural sensitivity toward sexual minorities emerge as constitutive elements of what is known as “affirmative therapy.” Though not a therapeutic model in itself, this is a therapeutic attitude that can result in specific interventions. Affirmative therapy has been defined as a culturally competent approach for working with LGBT patients, given that it stresses knowledge about LGBT topics and the use of the practical therapeutic skills mentioned above (Alessi et al., 2015; Boroughs et al., 2015; O’Shaughnessy and Speir, 2018; Pereira et al., 2019).

Although studies about the relationship between affirmative practice training and therapy outcomes are scarce, findings consistently reveal a positive association between these characteristics of therapists and some variables of the therapeutic process and its effects (Alessi et al., 2019).

### Process Components of the Psychotherapy With LGBT Patients

According to the Generic Model, all therapeutic approaches share six elements that constitute the core of the psychotherapeutic process (Orlinsky, 2009): (1) the therapeutic contract, which comprises the formal aspects of the process, including roles, duration, and involvement of third parties (e.g., parents and family); (2) therapeutic operations, during which the patient presents information and the therapist evaluates it and decides on the most suitable technical intervention; (3) the therapeutic bond, which comprises the relationship that the participants establish, including each person’s commitment to their role and the emotional, affective, and empathetic attunement present in their rapport; (4) participants’ openness or defensiveness, related to reflectivity and self-awareness in the therapeutic relationship; (5) intra-session impact, which involves therapeutic actions such as insight and emotional relief as well as (6) temporal patterns, which are part of session sequences throughout the process and can include intra-session micro-events and macro-events in each treatment phase.

Regarding the process components of psychotherapy with sexual and gender diverse people, the literature has shown – as will be exposed later – that the following are especially relevant to the experiences and behaviors of its participants: (a) the effects of psychotherapists’ prejudices and negative attitudes to LGBT patients, (b) the use of practices that affirm sexual and gender diversity in the sphere of psychotherapy, and (c) therapist self-disclosures when treating LGBT+ patients (see Figure 1B).

#### Effects of Psychotherapists’ Prejudices and Negative Attitudes to LGBT Patients

Until before 1970, clinical psychology research focused on the identification of the etiology of homosexuality and transgenderism and on the development of interventions for treating it and eventually curing it (i.e., conversion therapies; Clarke et al., 2010; Ryan et al., 2018). Consequently, for decades, all clinical models held a pathologizing view of diverse sexuality, thus contributing to the establishment of heteronormativity as a mental health ideal (Costa and Nardi, 2015). The academic training of several generations of psychologists and psychiatrists was informed by this perspective; therefore, they developed professionally in close contact with the beliefs and concepts promoted by said models. In this context, despite the progressive declassification of homosexuality as a psychopathological phenomenon, and given that nowadays most mental health professionals do not regard it as a disorder, practitioners continue to hold – sometimes inadvertently – beliefs and prejudices that result in actions that stigmatize sexual and gender diversity. These beliefs and attitudes are slow and hard to eradicate, since they are both implicit and deeply rooted in specific moral and cultural structures such as Catholicism, Christianity, and the traditional conception of family. Evidence provided by research on the attitudes to sexual diversity held by mental health professionals suggests that psychotherapists are not immune to biases, social prejudices, and the psychological stigmatization of sexual and gender diversity, being capable of inadvertently perpetuating them in their work with LGBT patients (Barrett and McWhirter, 2002; Bowers et al., 2005; Kilgore et al., 2005; Levounis and Anson, 2014; Bonamigo, 2016; Francia-Martínez et al., 2017).

Nowadays, open prejudices and explicit attacks on LGBT+ people are only found in some Middle East countries and within the context of reparative or conversion therapies (Byne, 2016; Ryan et al., 2018). In general, mainstream psychological and psychiatric ideas in the West tend to conceal implicit and unconscious prejudices and negative attitudes to LGBT people, which are sometimes held by well-intentioned psychotherapists who openly disagree with heteronormative practices and support the rights of sexual and gender diverse people (Alessi et al., 2015). These issues are known as microaggressions in the psychotherapeutic process (Sue, 2010; Shelton and Delgado-Romero, 2011). So, for instance, LGBT patients have reported discrimination, hostility, and negative therapeutic experiences characterized by subtle and covert microaggressions (Bowers et al., 2005; Greene, 2007; Israel et al., 2008; Shelton and Delgado-Romero, 2011); that is, seemingly harmless or meaningless psychotherapist utterances that – either consciously or unconsciously – convey mistaken beliefs, prejudices, and sexual stigmatization (Sue and Capodilupo, 2007). One of the described effects of these microaggressions in psychotherapy with LGBT patients is the silencing and invisibilization of diverse sexual and gender identity, which may exacerbate internalized sexual stigma in LGBT patients, reduce the therapeutic exploration of a wide range of experiences relevant to them, and heighten their hopelessness and depressive feelings (King et al., 2007; Shelton and Delgado-Romero, 2011). These effects can generate further psychological damage and be even more destructive than external and overt acts of discrimination and stigmatization (Speight, 2007), especially because microaggressions come from people who provide patients with psychological help and with whom they have established a trust-based relationship (Shelton and Delgado-Romero, 2011). In a study conducted in the United States by Shelton and Delgado-Romero (2011), LGBT patients identified seven microaggression categories: (a) the presupposition that sexual orientation is the cause of all the patient’s symptoms and conflicts, (b) avoidance and minimization of sexual orientation as a relevant area of exploration during psychotherapy, (c) the therapist’s attempts to over-identify him/herself with LGBT patients, (d) comments based on stereotyped assumptions about LGBT people, (e) expressions with a heteronormative bias, (f) the assumption that all LGBT people need psychotherapy, and (g) warnings about the potential harm derived from self-identifying as LGBT. All these forms of discrimination associated with sexual orientation and diverse gender identity, which may be present in psychotherapies with LGBT patients, reflect the ways in which beliefs and attitudes – grounded in social and cultural elements – negatively influence the psychotherapeutic process, constituting yet another factor in the mental health disparity (APA/Division 44, 2000; Cormier-Otaño and Davies, 2012; Hatzenbuehler et al., 2013; Levounis and Anson, 2014; Alessi et al., 2015; Bidell and Stepleman, 2017).

A survey of United States men and women who self-identify as bisexual revealed a high percentage of negative experiences with mental health professionals. Specifically, respondents stated that psychotherapists were ignorant of relevant aspects of bisexuality, criticizing and pathologizing their bisexual identities, also assuming that bisexuality is linked with conflicts and clinical issues (Page, 2004). Likewise, researchers surveyed 637 LGT people regarding what they expected from psychotherapy and their psychotherapists (Malley and Tasker, 2007). The participants reported that they appreciated the fact that their psychotherapists were knowledgable about aspects of diverse sexual identity and trusted them enough to discuss these subjects; also, they stressed how important it was for therapists to display an attitude of understanding and clinical listening unaffected by heterosexism (Lingiardi and Drescher, 2003). Regarding the latter aspect, Levounis and Anson (2014) note that mistaken beliefs and stereotypes are commonly present in psychotherapies with LGBT patients and can be reinforced both by patients and therapists. Thus, insufficient specific training, a lack of knowledge about LGBT culture and psychology, and scarce self-awareness of one’s beliefs and attitudes to sexual and gender diverse people can result in treatments in which patients experience microaggressions, thus confirming their fears and misgivings regarding psychotherapy and heightening their hopelessness regarding the help that they might receive. In other cases, this can cause the therapist’s prejudices and mistaken beliefs to complement the patient’s internalized sexual stigma, thus boosting the patient’s insecurities and anxiety about themselves (King et al., 2007).

#### Practices That Affirm Sexual and Gender Diversity in Psychotherapy

Over the last decades, affirmative psychotherapy approaches for LGBT patients have been developed; however, they do not constitute a psychotherapeutic model in itself but instead advocate for the inclusion of specific attitudes into psychological intervention connected with acceptance and understanding of the nature and challenges posed by sexual and gender diversity (Milton et al., 2002; Dillon et al., 2004; Kilgore et al., 2005; Bieschke et al., 2007; Martínez et al., 2018a). Authors have described a number of affirmative interventions aimed at actively depathologizing sexual diversity and cementing its place as a positive dimension of identity (Rock et al., 2010). Specifically, these practices lead to the creation of a safe and discrimination-free space, where psychotherapy with LGBT patients can be conducted. Generating such a space requires that psychotherapists be self-critical of their prejudices, use inclusive language, and openly display their affirmative position (Martínez et al., 2018a). Despite the importance of advocating for and developing affirmative psychotherapy, its guidelines are still too general and provide no specific pointers for therapeutic work focused on psychological difficulties common in LGBT people (e.g., internalized stigma, hypervigilance; Puckett et al., 2015), nor do they address psychological support in specific processes that sexual and gender diverse patients tend to display (e.g., revealing one’s diverse sexual orientation and transition to the gender identity felt).

#### Therapist Self-Disclosures in Therapeutic Work With LGBT+ Patients

Therapist self-disclosures (TSD) are verbal statements whereby the practitioner reveals something personal (Hill and Knox, 2002). Although these tend not to be common interventions in psychotherapy, research has shown that they have a positive impact on patients (Hill and Knox, 2003; Moore and Jenkins, 2012; Jeffery and Tweed, 2015). The scarce literature on this topic stresses that, used in moderation, TSDs can have a beneficial effect for patients, especially when they belong to a stigmatized population (Jeffery and Tweed, 2015). Feminist theories have supported the proper use of TSDs, noting that these interventions foster patient growth, create a feeling of solidarity between the therapist and the patient, generate a freer and more egalitarian relationship in terms of power, help the patient feel less embarrassed, and acknowledge the place of the real patient-therapist relationship (Mahalik et al., 2000). In general, this perspective highlights the fact that psychotherapy occurs within a biased social and historical context, and therapists are often culturally different from their patients; so therapists appreciate TSDs, asserting that these types of interventions encourage mutual trust and improve the therapeutic alliance (Sue and Sue, 1999; Moore and Jenkins, 2012). In this regard, as noted above, social changes and depathologization have increased sexual and gender minorities’ demand for mental health care, which poses new challenges for the majority of therapists who have never been trained to work affirmatively with LGBT+ clients (Bidell, 2016). Interventions such as TSDs could have beneficial effects by bringing about a culture of equality and recognition, regardless of therapists’ and patients’ sexual orientation or gender identity (Davies and Neal, 1996; Eubanks-Carter et al., 2005; Henretty and Levitt, 2010). In contrast, some studies have shown that concealment, especially in the case of gay or lesbian therapists working with heterosexual patients, generates strong discomfort and a state of permanent anxiety and ultimately has a negative impact on the relationship (Jeffery and Tweed, 2015). The main hypothesis about this phenomenon involves therapists’ prejudices and internalized homophobia, an under researched point that stands in contrast with the potential advantages of TSDs for the therapeutic process (Moore and Jenkins, 2012).

### Consequences of Psychotherapy With LGBT Patients

At this point, it is important to stress the difference between the outcomes and the consequences of psychotherapy. Outcomes are associated with a model that emphasizes aspects such as patient symptoms, personality traits, difficulties in interpersonal relationships, and a feeling of well-being. All these elements are relevant when evaluating the effectiveness or efficacy of a treatment, but provide a limited view of the effects that the psychotherapy can have on its participants. The consequences alluded to by the Generic Model include the aspects mentioned, but add the human context involved in the treatment, thus broadening the effect of psychotherapy (Orlinsky, 2009). Among other aspects, this model considers the impact not only on the patients’ family and work environment, but also on the therapist’s personal and professional life. In addition, it considers the effects that psychotherapy outcomes and/or the provision of psychotherapy can have on the service providing institution, in terms of economic, social, and cultural implications for the community to which it belongs, and on broader social aspects linked to the role of psychotherapy as a mental health tool.

Few studies have examined the clinical outcomes of psychotherapy with LGBT patients, let alone the consequences of psychotherapy for the life trajectories of its participants. On this subject, the studies and theoretical formulations that we have surveyed concern (a) patients’ satisfaction with the psychotherapy, (b) the consequences of positive and negative experiences in psychotherapy, and (c) the construction of a positive identity and a legitimate life plan (see Figure 1C).

#### Patients’ Satisfaction With Psychotherapy

A number of studies suggest that the psychological and psychotherapeutic care does not seem to meet the specific needs of LGBT people (Budge et al., 2017). For instance, a study conducted in New York city found that 17.6% of LGBT participants, compared to 8% of heterosexual participants, reported being dissatisfied with the mental health care received (Avery et al., 2001). This situation is more severe for transgender people. For instance, an online survey conducted by Simeonov et al. (2011) revealed that over 50% of LGB people and/or those who self-identified as trans did not think that their mental health care needs were being met and, specifically, that trans people had stopped requesting these services due to negative experiences. Similarly, in a qualitative study that we conducted in Chile with young LGBT people who had undergone a suicidal process, participants reported that the help provided by the adult world – both school counselors and psychotherapists in mental health services – was clumsy and sometimes compounded their problems (Tomicic et al., 2018).

#### Consequences for Patients of Positive and Negative Experiences in Psychotherapy

Regarding the consequences of being exposed to positive and negative psychotherapeutic interventions, a study conducted in the United States on the experiences of LGBT patients in psychotherapy (Israel et al., 2008) provides interesting findings. The authors concluded that positive actions and interventions – a therapeutic relationship characterized by trust, acceptance, and an affirmative attitude by the therapist – led to improved quality of life, a better relationship with the therapist, more self-awareness, self-acceptance and/or willingness to change, and the development of a positive sexual and gender identity. In contrast, negative actions and interventions – when therapists are perceived as cold, distant, and prejudiced people who impose their views and perform microaggressions – had a negative impact on the therapeutic relationship, in most cases leading to premature termination. In addition, these experiences had a negative influence on patients’ quality of life; also, for those who stayed in therapy, they kept them from revealing or exploring issues related to their reasons for seeking help, generated a negative impact on the process of affirming their diverse sexual and/or gender identity, and caused them to develop a negative overall impression of psychotherapy (Israel et al., 2008).

#### Psychotherapy With LGBT Patients and Construction of a Positive Identity and a Legitimate Life Plan

Regarding the overall consequences and outcomes of psychotherapy, constructing a positive identity and being able to envision a feasible life plan emerge as two of the key changes experienced by LGBT patients (Proujansky and Pachankis, 2014; Martínez et al., 2018a). These achievements that psychotherapy facilitates are not different from those experienced by patients who are not sexual or gender diverse (e.g., Krause et al., 2018). However, in the case of LGBT people, this involve a shift from accepting their diverse sexual and gender identity to affirming their sexuality as a healthy and gratifying aspect of their overall identity (Proujansky and Pachankis, 2014; Tomicic et al., 2018). This shift is a key, since self-alienation and postponing the development of a positive identity, due to the encouragement of various levels of concealment or invisibilization and the internalization of sexual stigma, can lead to a progressive decrease in self-esteem, isolation, and suicidal behaviors (Savin-Williams, 2001). Thus, for instance, a retrospective study on the experiences of LGBT adolescents and young people regarding suicide (Tomicic et al., 2018) revealed that participants had a permanent feeling of hopelessness linked to the impossibility of constructing a positive identity and finding a legitimized place in their family, interpersonal, and/or social life. In this context, Proujansky and Pachankis (2014) suggest that the psychotherapy should help patients not only to accept their diverse sexual and/or gender identity, but also to learn to recognize themselves as part of a historical legacy (e.g., by promoting activism, volunteering, creativity, and pride) as well as find and construct relationships and communities that help them to overcome the isolation and depression while developing self-affirmative communication skills (Lelutiu-Weinberger and Pachankis, 2017).

## Discussion

This article argues for the use of the Generic Model of Psychotherapy (Orlinsky and Howard, 1987) as an organizing framework for psychotherapy research with sexual and gender diverse patients. We have illustrated some of the main components offered by the Generic Model, proposing a selection of those specific to research in the field of sexual minorities and stressing areas marked by knowledge gaps that require future developments. In this regard, we have observed that research in the field of the *determinants* of psychotherapy has focused on the persistence and emergence of new forms of victimization – e.g., the effects and expressions of internalized stigma – and on the identification of mental health disparities and barriers to psychological treatment for LGBT+ people (e.g., psychotherapist training and specialization). We believe that, in this field, it may be important for researchers to connect the evolution of social and political contexts (e.g., gender identity laws and non-discrimination due to sexual and gender orientation) with their effects on the beliefs and prejudices held by both therapists and patients regarding psychological help. For instance, this could be tackled by asking questions about patients and therapists’ views on sexual and gender diversity and the ways in which these beliefs generate barriers to access and/or a positive attitude to psychotherapy; likewise, questions could be asked about the negative and positive expectations of LGBT people regarding psychotherapy, about the sources of these expectations, and about how they hinder or facilitate the search for psychotherapeutic help.

With respect to the psychotherapy process components, research has focused on aspects that directly affect the positive or negative course of the therapeutic relationship. Studies on affirmative therapy have not yielded evidence about more specific tools for working with internalized stigma or hypervigilance, rather, they have focused on examining the creation of a safe space for patients. The interesting and novel research on the effects of TSDs on patients still requires further details about the effects of these interventions on therapists. An interesting step in this field would be to study internalized stigma in LGBT+ therapists and its effects on psychotherapy and patients’ change process. We propose several questions in this regard: how are topics related to gender identity and/or sexual orientation integrated into (a) the construction of the patient’s mental health problem, his/her/their reasons for seeking help, and the trajectory of the psychotherapeutic process? (b) how do the impact and role of internalized stigma and hypervigilance in LGBT patients and therapists influence the establishment of the trust relationship and a positive therapeutic alliance? (c) how do microaggressions generate ruptures and difficult moments in the psychotherapeutic process? and (d) how do therapists’ and LGBT patients actions facilitate the resolution of such ruptures?

Lastly, regarding the consequences of psychotherapy, research has tended to focus on immediate, short-term effects on patients. Very few studies have delved deeper into the effects of psychological help on patients’ later life, taking into account not only their symptoms, but also the construction of their social identity. Likewise, authors have been slow to examine the effects of psychotherapy on patients’ familial and social relationships. Even fewer studies have sought to determine how working with LGBT+ patients requires a specific type of academic training for psychotherapists and how this can affect the professional culture of the health care system providing psychological help in a specific context. Some questions that we propose for new developments in this field concern the interventions and actions of psychotherapists that have a positive influence on the change process, the aspects of psychotherapy that LGBT patients take into account in their overall assessment of the psychotherapeutic process, the negative consequences of psychotherapy that patients identify, and the changes that therapists see in themselves due to conducting psychotherapeutic processes with LGBT patients.

Regarding clinical implications that can be tackled by applying the Generic Model to the practice of psychotherapy with patients of sexual and gender minority groups, three core aspects associated with each one of the main components of the model could be stressed (see Figure 1). In the first place, practitioners should always keep in mind that part of the LGBT patients’ symptoms and mental health condition could be determined by the high or low presence of stigma, prejudice, and discrimination associated with sexual and gender diversity; these and other possible determinants could be actively explored or incorporated into their case formulations. Second, a central aspect of the therapeutic work with LGBT patients should be directed at the identification and intervention of internalized stigma. In this regard, practitioners should pay special attention to possible microaggressions present in their interventions, which could reinforce the sexual stigma of their patients; on the contrary, from the beginning, the practitioner should focus their efforts on characterized by an affirmative supportive attitude, in a safe space. Third, practitioners should always keep in mind that psychotherapy’s main contribution for LGBT patients is not only related to the lessening of symptoms and discomfort, but also the strengthening of their identity, promoting the development of a valid, hopeful life plan.

The search of the literature discussed in this article was not exhaustive; thus, we may have failed to discuss some studies that tackle the issues that we propose. Nevertheless, the exploration performed enabled us to illustrate research on sexual and gender diversity based on the organizing guidelines provided by the Generic Model of Psychotherapy, which was our main goal. This task also made it clear how much there is left to study in this field and foregrounds a model that, despite its age, remains an excellent map for guiding psychotherapy researchers through the various domains that comprise human psychology.

## Author Contributions

AT, CM, and JR participated in the intellectual conception and review of the literature. AT and CM prepared the manuscript. JR contributed to the discussion and review of the coherence of the article. All authors contributed to the article and approved the submitted version.

### Conflict of Interest

The authors declare that the research was conducted in the absence of any commercial or financial relationships that could be construed as a potential conflict of interest.
